# Supplementary material for: Fouling Mitigation by Optimizing Flow Rate and Pulsed Electric Field during Bipolar Membrane Electroacidification of Caseinate Solution
Source: Membranes (Basel). 2021 Jul 15;11(7):534. doi: 10.3390/membranes11070534 (PMC8307857; doi:10.3390/membranes11070534)
Supplement: Supplementary file 1 [file membranes-11-00534-s001.zip › membranes-1296572-supplementary.pdf]

Supplementary materials

Table 1S. Membrane properties (thickness and conductivity) before and after EDBM carried out in different conditions of flow rate (corresponding to indicated Reynolds numbers) and PEF current mode with pulse-pause durations of 10 s - 10 s, 10 s - 20 s, 10 s – 33 s and 10 s - 50 s. Reynolds numbers of 187, 374 and 560 correspond to flow rates of 7.8, 15.6 and 23.4 cm/s respectively.

**Current mode**			**10 s–10 s**			**10 s-20 s**			**10 s-33 s**			**10 s-50 s**		
**Reynolds number**			**187**	**374**	**560**	**187**	**374**	**560**	**187**	**374**	**560**	**187**	**374**	**560**
Conductivity (mS/cm)	CEM	Before	8.87 ± 0.46 a	9.01 ± 0.29 a	8.39 ± 0.54 a	8.58 ± 0.42 a	8.99 ± 0.14 a	8.87 ± 0.37 a	9.02 ± 0.24 a	8.64 ± 0.42 a	8.64 ± 0.24 a	9.32 ± 0.14 a	8.66 ± 0.42 a	9.17 ± 0.40 a
		After	7.99 ± 0.49 bA	8.05 ± 0.31 bA	8.01 ± 0.46 bA	8.20 ± 0.31 bA	8.08 ± 0.09 bA	7.90 ± 0.10 bA	8.11 ± 0.38 bA	7.73 ± 0.40 bA	7.93 ± 0.46 bA	8.63 ± 0.31 bB	8.05 ± 0.24 bB	8.15 ± 0.21 bB
	BPM 1	Before	6.02 ± 0.89 a	5.51 ± 0.72 a	5.12 ± 0.38 a	4.86 ± 1.23 a	4.97 ± 0.76 a	4.97 ± 0.60 a	5.23 ± 1.14 a	5.47 ± 0.21 a	4.69 ± 0.63 a	5.54 ± 1.01 a	4.53 ± 0.14 a	5.11 ± 0.98 a
		After	6.49 ± 0.54 b	5.96 ± 0.27 b	6.13 ± 0.15 b	6.32 ± 0.39 b	5.83 ± 0.29 b	5.61 ± 0.44 b	5.95 ± 0.48 b	6.13 ± 0.42 b	5.61 ± 0.39 b	5.99 ± 0.48 b	5.91 ± 0.29 b	5.54 ± 0.51 b
	BPM 2	Before	5.46 ± 0.91 a	5.44 ± 0.48 a	4.33 ± 1.27 a	5.52 ± 1.09 a	4.03 ± 0.90 a	4.82 ± 1.43 a	4.93 ± 0.22 a	4.31 ± 0.54 a	5.00 ± 0.90 a	4.41 ± 0.44 a	5.98 ± 0.68 a	5.03 ± 1.10 a
		After	6.22 ± 0.71 b	6.23 ± 0.30 b	5.41 ± 1.17 b	6.61 ± 0.59 b	5.55 ± 0.91 b	6.21 ± 0.65 b	6.13 ± 0.01 b	5.82 ± 0.50 b	6.21 ± 0.47 b	5.86 ± 0.44 b	6.65 ± 0.56 b	5.87 ± 0.68 b
Thickness (mm)	CEM	Before	0.151 ± 0.002 a	0.154 ± 0.005 a	0.152 ± 0.009 a	0.149 ± 0.009 a	0.148 ± 0.005 a	0.155 ± 0.009 a	0.154 ± 0.003 a	0.156 ± 0.005 a	0.148 ± 0.006 a	0.153 ± 0.012 a	0.153 ± 0.003 a	0.154 ± 0.008 a
		After	0.151 ± 0.010 a	0.151 ± 0.002 a	0.156 ± 0.003 a	0.154 ± 0.005 a	0.149 ± 0.006 a	0.155 ± 0.009 a	0.154 ± 0.001 a	0.158 ± 0.004 a	0.151 ± 0.005 a	0.157 ± 0.012 a	0.160 ± 0.003 a	0.152 ± 0.007 a
	BPM 1	Before	0.245 ± 0.005 a	0.245 ± 0.002 a	0.240 ± 0.006 a	0.243 ± 0.004 a	0.245 ± 0.004 a	0.244 ± 0.006 a	0.249 ± 0.006 a	0.246 ± 0.002 a	0.243 ± 0.004 a	0.241 ± 0.004 a	0.238 ± 0.002 a	0.240 ± 0.006 a
		After	0.245 ± 0.003 a	0.239 ± 0.011 a	0.242 ± 0.004 a	0.263 ± 0.025 a	0.244 ± 0.009 a	0.247 ± 0.003 a	0.250 ± 0.005 a	0.241 ± 0.009 a	0.243 ± 0.005 a	0.243 ± 0.006 a	0.243 ± 0.002 a	0.242 ± 0.010 a
	BPM 2	Before	0.243 ± 0.003 a	0.249 ± 0.003 a	0.235 ± 0.007 a	0.245 ± 0.007 a	0.239 ± 0.005 a	0.244 ± 0.002 a	0.240 ± 0.006 a	0.237 ± 0.006 a	0.242 ± 0.010 a	0.236 ± 0.013 a	0.241 ± 0.002 a	0.243 ± 0.002 a
		After	0.244 ± 0.012 a	0.245 ± 0.002 a	0.239 ± 0.006 a	0.251 ± 0.007 a	0.237 ± 0.006 a	0.244 ± 0.002 a	0.238 ± 0.004 a	0.239 ± 0.005 a	0.243 ± 0.006 a	0.237 ± 0.004 a	0.242 ± 0.001 a	0.242 ± 0.004 a

*Data marked with different letters (a, b or A, B) differ significantly from each other; lowercase letters indicate differences in conductivity and thickness before and after ED process for the same membrane; uppercase letters indicate differences between the electric current modes for the same membranes
